# Data of pre-IPO or listing firm characteristics and post-listing stock returns of Chinese listed firms

**DOI:** 10.1016/j.dib.2020.105226

**Published:** 2020-01-31

**Authors:** Hong Li

**Affiliations:** University of Nottingham, United Kingdom

**Keywords:** Overseas listing, Pre-IPO firm characteristics, Post-listing stock returns

## Abstract

The data set contains the data of variables, such as indicators of forms or host markets of the Chinese listed firms, pre-IPO or listing firm characteristics and post-IPO or listing stock returns. These variables can be used to estimate the probabilities of overseas listing in specific forms or locations through binary probit or multinomial logistic regression, and to evaluate the consequences of overseas listing in some specific forms or locations via the two-factor asset pricing model. Furthermore, these data can be used to estimate the models simultaneously within the potential outcome framework as done in the paper, entitled ‘Direct overseas listing versus cross-listing: A multivalued treatment effects analysis of Chinese listed firms’. https://doi.org/10.1016/j.irfa.2019.101391.

**Table:** Specifications Table

Subject	*Finance*
Specific subject area	*Asset- pricing model*
Type of data	*Excel spreadsheet*
How data was acquired	*Raw data are either downloaded from websites of HKSE, NYSE and NASDAQ; French-Fama data library; China Security Market and Accounting Research databases, Datastream, World Bank data bank, and Chinese statistical yearbooks or hand-collected by reading the IPO prospectus.*
Data format	*Raw and processed for estimation*
Parameters for data collection	*Overseas-listed firms must be on the official list provided by Chinese Securities Regulatory Commission.* *Pre-IPO or listing financial data are available on their prospectus or in CSMAR databases.* *Post-listing stock returns are available for at least 36 months.*
Description of data collection	*Pre-IPO or listing data for overseas listed firms are hand-collected by reading the prospectus filed with HKSE, NYSE or NASDAQ.* *Pre-IPO or listing data of domestically listed firms are downloaded from CSMAR.* *Post-listing share prices, risk-free rates and market indices are also downloaded from CSMAR.* *Post-listing provincial and national economic variables are obtained from Chinese Statistical Yearbooks or World Bank data bank.* *US returns and risk-free rates are obtained from French-Fama data library.* *Hong Kong market returns and risk-free rate are downloaded from Datastream.*
Data source location	*Websites of HKSE, NYSE and NASDAQ; French-Fama data library; Datastream, China Security Market and Accounting Research databases, World Bank data bank, and Chinese statistical Yearbooks.*
Data accessibility	*Data is with this article.*
Related research article	*Li, H. Direct overseas listing versus cross-listing: A multivalued treatment effects analysis of Chinese listed firms, International Review of Financial Analysis, 2020 (forthcoming)* [1]

**Table:** 

**Value of the Data** • These data are unique, as pre-IPO firm characteristics are hand-collected for the 243 Chinese firms that first listed overseas during 1997–2015. • Researchers on listing issues, such as listing motivations and consequences, can benefit from these data. • The data *could be compared with other data for further insight or serve as a benchmark for other researchers.*

## 1 Data description

There are two spreadsheets in this data supplement file. The sample size is the same across two sheets at 1688 purely domestically listed firms and 243 overseas listed firms. While the pre-IPO or listing firm characteristics are the same, the data of excess returns of individual assets, Chinese national market and global market differ between two sheets. In one sheet, the short-run stock returns and market returns are presented. These are stock or market returns within 6 months of first listing, either domestically or overseas. In the other sheet, the long-run stock or market returns are included. These are calculated for the period of the 7th month up to 36th month posting listing.

## 2 Experimental design, materials, and methods

A list of Chinese overseas listed firms was downloaded from the website of the Chinese Security Regulatory Commission. The names of these firms and their listing years are checked against those reported on the websites of NYSE and HKSE. To identify the forms of overseas listing by these companies, I have checked these firms' names and listing years against the records of domestically listed firms in the China Stock Market and Accounting Research Database (CSMAR). On the basis of these checks, four forms of overseas listing are identified for the Chinese overseas listed firms. They are purely overseas listed firms, firms that simultaneously listed domestically and overseas, conventionally cross-listed firms (domestically listed firms that subsequently listed overseas), and firms that cross-listed in reverse order (overseas-listed firms that subsequently listed domestically). Furthermore, the host markets, the US and/or Hong Kong, of these overseas listed firms are also identified. Indicators are assigned to these firms by their listing form and host market. The raw data are downloaded from the databases such as CSMAR or hand-collected by reading firms' prospectuses filed on the websites of NYSE or Hong Kong stock exchange. Calculation is made via Excel to form the pre-IPO or listing and post-IPO or listing firm characteristics and short- and longer-run post-listing stock returns.
